# Air pollution and general practitioner access and utilization: a population based study in Sarnia, 'Chemical Valley,' Ontario

**DOI:** 10.1186/1476-069X-10-71

**Published:** 2011-08-09

**Authors:** Tor H Oiamo, Isaac N Luginaah, Dominic O Atari, Kevin M Gorey

**Affiliations:** 1 Department of Geography, The University of Western Ontario, London, Ontario, Canada; 2 Department of Geography, Nipissing University, North Bay, Ontario, Canada; 3 School of Social Work, University of Windsor, Ontario, Canada

## Abstract

**Background:**

Health impacts of poor environmental quality have been identified in studies around the world and in Canada. While many of the studies have identified associations between air pollution and mortality or morbidity, few have focused on the role of health care as a potential moderator of impacts. This study assessed the determinants of health care access and utilization in the context of ambient air pollution in Sarnia, Ontario, Canada.

**Methods:**

Residents of Sarnia participated in a Community Health Study administered by phone, while several ambient air pollutants including nitrogen dioxide (NO_2_), sulphur dioxide (SO_2_) and the volatile organic compounds benzene, toluene, ethylbenzene, mp- and o-xylene (BTEX) were monitored across the city. Land Use Regression models were used to estimate individual exposures to the measured pollutants and logistic regression models were utilized to assess the relative influence of environmental, socioeconomic and health related covariates on general practitioner access and utilization outcomes.

**Results:**

The results show that general practitioner use increased with levels of exposure to nitrogen dioxide (NO_2_- Odds Ratio [OR]: 1.16, *p *< 0.05) and sulphur dioxide (SO_2_- OR: 1.61, *p *< 0.05). Low household income was a stronger predictor of having no family doctor in areas exposed to high concentrations of NO_2 _and SO_2_. Respondents without regular care living in high pollution areas were also more likely to report travelling or waiting for care in excess of 20 minutes (OR: 3.28, *p *< 0.05) than their low exposure counterparts (OR: 1.11, *p *> 0.05).

**Conclusions:**

This study provides evidence for inequitable health care access and utilization in Sarnia, with particular relevance to its situation as a sentinel high exposure environment. Levels of exposure to pollution appears to influence utilization of health care services, but poor access to primary health care services additionally burden certain groups in Sarnia, Ontario, Canada.

## Background

Research based on recent conceptualizations of health that recognize socioeconomic and environmental determinants in Canada shows that significant health disparities continue to persist, despite a health care system based on the premise of universal and equitable access [1,2]. Regional health care expenditures in Ontario are also associated with toxic pollution output [3,4]. Such associations between the physical environment and health have highlighted the need to study the compounding impacts of environmental and socioeconomic stressors with a focus on context specific environmental health and health care issues.

Analyses of primary health care outcomes under the assumption of universal access have revealed that access and utilization depend on a host of individual, social and environmental factors. For example, physician use is determined by both individual and neighbourhood income in addition to educational attainment [5,6]. Dunlop et al. [7] found that lower socioeconomic status (SES) was associated with increased use of primary care and lower rates of specialist service utilization, whereas health needs as measured by perceived health status and health condition predicted utilization across the board. Finkelstein [8] found that health care expenditures were related to income, but this association disappeared after adjusting for health status. Curtis and MacMinn [9] indicated that inequities related to lower levels of income and education in Canada actually grew between 1978 and 2003, while health status remained strongly associated with health care utilization. However, SES inequities in utilization appeared attenuated after initial contact with the primary care system. Among studies that did not consider temporality, but rather focused on spatiality, efforts have included controlling for health service environments as different among Public Health Units [10], and both system- and individual- related barriers within neighbourhoods [11].

Birch et al. [12] argued that health care resource allocation should be based on the needs of a particular community rather than presenting patients, who in practicality form the basis for funding to a particular service provider. This stems from provider inability to determine the needs of those who do not present themselves as patients, which is a problem reflected in the geography of health care literature concerned with the spatial organization of health services. Geographic analysis of health care draws attention to the complexity of population needs in health service access and utilization [13]. The argument is that the relationships between individual, population and neighbourhood characteristics that determine need in a particular context (e.g., demographics, SES, mobility, ethnicity, pollution, etc.) need to be examined in order to understand health care issues. Equitable access to health care cannot be meaningfully discussed without considering needs [14].

Research on health care outcomes with explicit reference to the physical environment is most often conducted at the level of neighbourhood or intra-urban scales to capture adequate resolution of spatial variability [15,16]. Previous studies have established relationships between neighbourhood and population characteristics that amplify environmental health impacts, but to our knowledge no studies have examined the relationship between health care and air quality directly. However, existing literature indicates that people exposed to higher levels of air pollution are particularly vulnerable to the effects of inequitable access to primary health care [17]. Access to primary care helps prevent illness and death, and is also more highly associated with equitable distribution of health in populations than specialty care [18]. Gwynn and Thurston [19] suggest that disparate access to health care may increase susceptibility to the effects of air pollution. In a Canadian context, this is of particular interest since the Canada Health Act implicitly assumes that care is available to those who are in need, but individual providers have historically been trusted to allocate resources according to federal objectives [12]. Local health care providers are faced with resource restraints that can make it difficult to meet federal objectives, and in this study we seek to further understand how the distribution of air pollution is associated with consumption of health care in Sarnia, Chemical Valley, Ontario [20].

### Study Context

**Figure 1:**
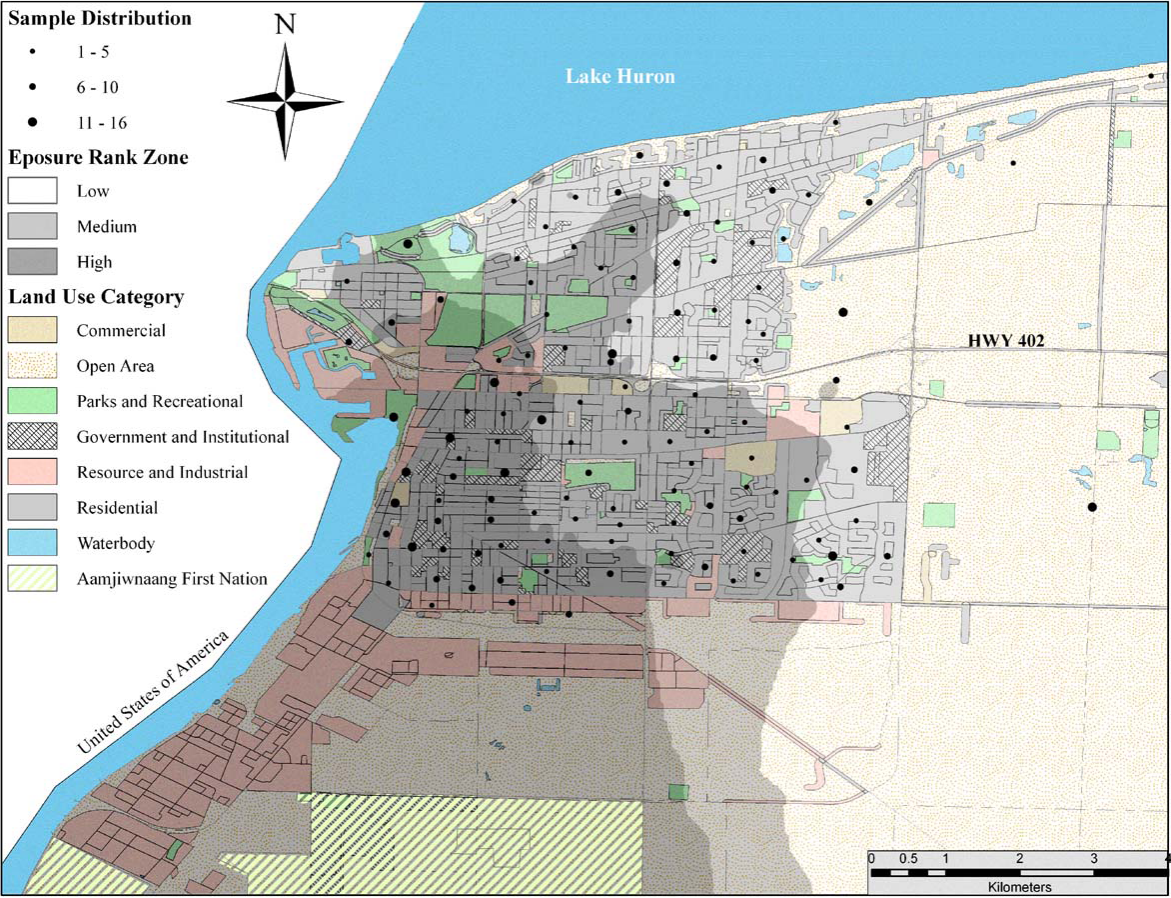
**Inverse Distance Weighted interpolation surface of respondent exposure rank tertiles based on NO_2 _and SO_2_**. Distribution of respondents displayed at centroid of census dissemination areas (DA) with symbol size representing the number of respondents in the DA.

The City of Sarnia has a population of 71,419 and covers approximately 800 km^2 ^[21]. The Sarnia area is called 'Chemical Valley' as it is home to more than 40 per cent of all chemical processing facilities in Canada. Sarnia is also located within the government designated St. Clair River Area Of Concern (AOC), which among 16 other areas in Canada was declared in further need of health investigations relating to impacts of environmental pollution [22]. The region is also subject to significant outputs of vehicular exhaust due to the Canada-US international crossing at Bluewater Bridge, and transnational air and water pollution from Ohio, Illinois and Michigan. The Ontario Medical Association [4] estimated that Sarnia and the surrounding Lambton County suffered 100 premature deaths, 270 hospital admissions, 920 emergency visits and 471 700 minor illness days due to air pollution in 2005 alone. Fung and colleagues [20] showed that rates of hospitalization were significantly higher in Sarnia than Windsor and London, two nearby cities in Southwestern Ontario. Furthermore, a study focused on the Aamjiwnaang First Nation's reserve, which is surrounded by Chemical Valley (Figure 1), reported a sex ratio of 2:1 in favour of females and attributed this anomaly to accumulative effects of pollution [23,24]. This background information calls for the need to examine the relationship between air pollution and health care utilization at the community level. The findings will provide guidance to local level health promotion and preventive care deficiencies with a focus on areas with poor air quality.

## Methods

We use a combination of spatial, environmental, and survey data to generate and test the hypothesis that relationships between air pollution and determinants of health, access and utilization of general practitioners (GP) do exist in Sarnia, Ontario. Residents of Sarnia took part in a community health study in 2005. A stratified random sampling procedure was used to select respondents from each census tract. The survey was conducted by Canadian Viewpoint Ltd, a survey company in Toronto, Ontario (http://www.canview.com) using a computer assisted telephone interview system and introduced as a general health survey. The sample represented approximately 1% of the Sarnia population, yielding a total of 804 respondents with a response rate of 62%. This sample size was determined to be sufficiently large for the number of variables included in the analyses [25]. The study was approved by the Social Sciences Research Ethics Board at The University of Western Ontario.

### Outcome Variables

Access to health care was measured with the question "Do you have a regular family medical doctor or health care provider?" We recognize that health care consumers have over the last decade become more dependent on primary care sources other than family doctors (e.g., nurse practitioners, walk-in clinics). Hence this conceptualization of access may be a limited measure of the potential for health care use. Nonetheless, access to a regular medical doctor is supposed to be equitable and represents a particular aspect of quality of care, because continuity of regular care can strongly influence health care satisfaction and use of health care services [26]. Furthermore, based on the important distinction between this definition of access versus the act of using or receiving health care [27], we measure utilization of primary health care, or general practitioner (GP) use, by a positive response to the question "in the past two weeks, have you seen or talked on the telephone with your family doctor about your physical, emotional or mental health?" Similar questions have been used in previous health care utilization studies [16], and Roberts et al. [28] showed that that this self-reported measure is reasonably accurate. Therefore we used these questions to evaluate short-term effects of air pollution on health care utilization and equitable access to services, and furthermore identify the inherent mediating factors that contribute to both.

### Independent Variables

#### Exposure Assessment

During administration of the survey several ambient air pollutants including nitrogen dioxide (NO_2_), sulphur dioxide (SO_2_) and the volatile organic compounds benzene, toluene, ethylbenzene, mp- and o-xylene (BTEX) were monitored at 39 locations across the city of Sarnia for 2 weeks in October 2005 [29,30]. Land use regression (LUR) was utilized to model the ambient NO_2_, SO_2 _and total BTEX concentrations because of its potential to provide spatial pollution estimates in small-areas without the data requirements and related expenses incurred in other exposure modelling techniques, such as dispersion [31] and micro-environmental monitoring [32]. LUR modelling uses nearby traffic, land use and population variables to explain the spatial variability of air quality [33]. For a comprehensive review of LUR models for exposure assessment in epidemiologic studies see Hoek et al. [34]. We controlled for personal exposure by asking the respondent what number of outdoor (gases, dusts, fumes, and pesticides) or indoor (pets, carpets, rugs and fireplaces) irritants to which they were regularly exposed. Table 1 provides a description of all variables that were included in the analysis.

**Table 1:** Explanatory variables in final logistics model

Variable	Type	Coding
***Demographic***		

Age of respondent	Categorical	18-24* vs 25-44, 45-64, 65+

Gender	Categorical	Male* vs Female

***Exposure***		

Nitrogen dioxide (NO_2_)	Continuous	

Sulphur dioxide (SO_2_)	Continuous	

Benzene, toluene, ethylbenzene and xylenes (BTEX)	Continuous	

Gas, dust, fumes and pesticides (outdoor exposure)	Continuous	

Pets, carpets, rugs and fireplace (indoor exposure)	Continuous	

***Socioeconomic***		

Marital status	Categorical	Partner* vs No partner

High school graduate (education)	Categorical	Yes* vs No

Below Low Income Cut Off	Categorical	No* vs yes

Employment status	Categorical	Employed*, Other, Unemployed

Children under 18 (children)	Categorical	No* vs yes

Housing tenure	Categorical	Owned* vs rented

Housing condition	Categorical	Sastisfactory* vs needs repair

***Health Behaviour***		

Frequency of monthly alcohol use	Continuous	

Regular smoker	Categorical	No* vs Yes

Hours of weekly exercise	Continuous	

Voluntary Medical Check-up	Categorical	No* vs Yes

***Community & Environmental Perception***		

Odour annoyance	Continuous	0-10

Aware that Sarnia is considered in an Area of Concern (awareness)	Categorical	No* vs Yes

Believe odours/pollution cause health problems (pollution health)	Categorical	No* vs Yes

Community satisfaction	Categorical	Dissatisfied* vs Satisfied

***Health Care***		

Health care provider travel and wait times (Care availability)	Categorical	≤ 20 min* vs > 20 min

***Health Status***		

Physician diagnosed chronic health problems (chronic conditions)	Continuous	

Health problems from pollution-induced stress (stress symptoms)	Continuous	

Cardinal health problems of pollution (cardinal symptoms)	Continuous	

Back- or joint-pain and easy bruising (control symptoms)	Categorical	None* vs any

Above cut-point 4 positive responses on General Health Questionnaire	Categorical	No* vs Yes

Self-rated health compared to other people same age	Categorical	Good or better* vs Fair/poor

Ability to cope with daily problems (coping)	Categorical	Good or better* vs Fair/poor

***Outcomes***		

***Health Care Access***		

Regular health care provider or family physician (GP Access)	Categorical	Yes* vs No

***Health Care Utilization***		

General practitioner use in the past 2 weeks (GP Use)	Categorical	No* vs Yes

* Reference category.

#### Community Context

Respondents were asked to assess their degree of annoyance due to air pollution odours on an 11-point scale as similar measures have previously been used to study annoyance [35]. Respondents were also asked if they believe the odours from the chemical plants were harmful to their health and whether they were aware of Sarnia being classified as an AOC. These were dichotomous variables with those who answered "no" used as the reference categories. A categorical variable constructed from travel and wait times for health care provided a measure of availability and those who responded with "20 minutes or less" for either waiting or traveling were used as the reference category [16]. Respondents were also asked to rate their level of satisfaction with Sarnia as a community. The variable was dichotomized with the first two responses of the following question defined as the outcome of interest - "In general, how satisfied are you with your community as a place to live? Would you say you are Very Satisfied, Somewhat Satisfied, Not Too Satisfied, or Not At All Satisfied?"

#### Health and Behaviour

Chronic health was included as a continuous variable of the number of conditions diagnosed by a physician and included skin conditions, respiratory disease, hypertension and cancer. General symptoms were defined as those likely to be caused by stress-mediated mechanisms of pollution and included chest pains, headaches, dizzy spells, sleep problems, stomach aches, diarrhea and loss of appetite. Cardinal symptoms were defined as those likely to be the result of irritant properties of air pollution and included coughs, wheezing/breathing problems, nausea, sinus congestion, colds, skin rashes, eye, nose or throat irritations, earaches and nosebleeds [36]. Control symptoms (backpain, jointpain and easy bruising) not likely related to air pollution were also included. Mental health was measured using the General Health Questionnaire (GHQ - 20 items) [37]. Those who provided positive responses to at least four of the questions were categorised as emotionally distressed [38]. Respondents' self-rated health compared to others their age was classified as poor or fair versus good, very good or excellent. Coping was classified the same way as health status, but by response to the question "how would you rate your ability to handle day-to-day demands in your life?" Measures of health related behaviour including smoking, alcohol consumption, exercise and medical check-ups were also included.

#### Socioeconomic and Demographic Characteristics

Age was included as a categorical variable with the intervals 18-24, 25-44, 45-64, and 65 or older, while males were used as the reference category for the influence of sex. Income below the Statistics Canada Low Income Cut-Off [39] at $22 139 before tax for the median number of household members in our sample and population size in Sarnia, having no children under age 18, marital status, completion of high school and employment were used as reference categories for variables measuring family characteristics and SES. Housing condition was coded as "satisfactory" vs. "in need of repair", while housing tenure was coded as "owning" vs. "renting". These housing variables were included in the analysis to control for their potential influence on health and as an additional SES measure (e.g., [40]).

### Analysis

Binary logistic regression models for the dependant variables were built in which each consecutive, conceptually grouped, block of variables were entered into a stepwise regression algorithm (SES, health and behaviour, community context, and pollution health effects) following forced entry of *a priori *variables. The *a priori *variables were chosen because of their influence as demonstrated in previous studies and included age, sex, family doctor, chronic disease and mental health in the GP use model [41] and income, age, sex, unmet health care needs in past 12 months and self rated health [11,42] in the GP access model. The variables that made a significant contribution to the model at the entry of each block were retained and included in the next block. As suggested by Hosmer and Lemeshow a less stringent significance level of 0.15 for inclusion was utilized to ensure variables with coefficients different from zero were included [43].

**Figure 2:**
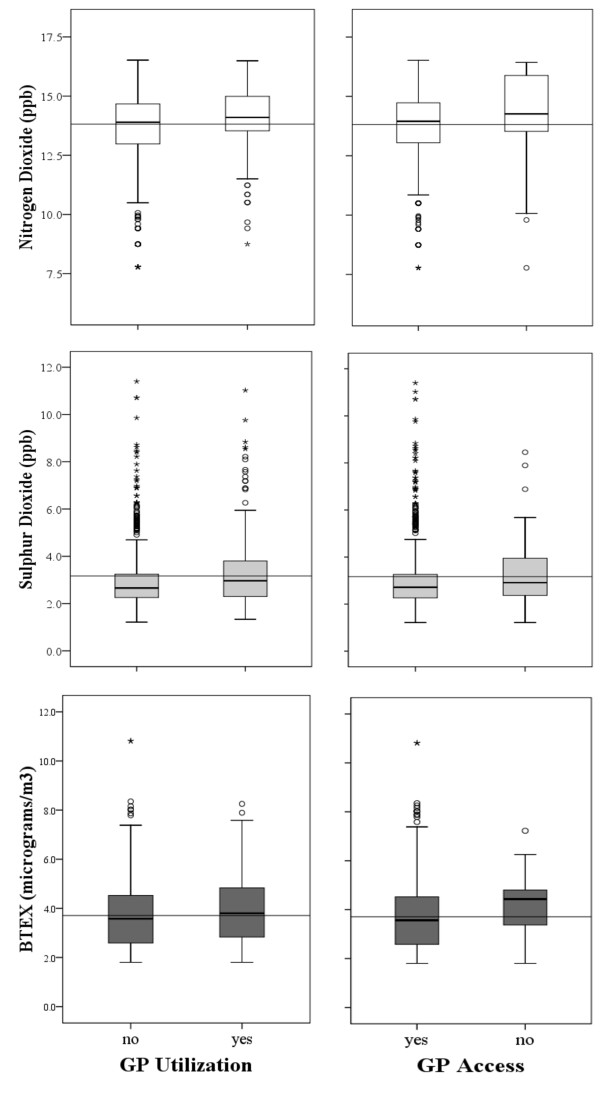
**Box-plot diagrams of NO2, SO2, and BTEX values for respondents based on Land Use Regression Models within categories of general practitioner (GP) utilization and regular GP Access**. Solid lines across panels represent pollutant average and inside boxes the median. (○): 1.5-3 times Interquartile range; (★) 3 or more times interquartile range.

Based on our study objectives, we examined if burdens of air pollution were borne by residents of Sarnia who were also at a disadvantage with respect to health care access. Therefore, *a priori *and other significant independent variables remaining in the final models for GP use and access were forced into regression models that assessed the impacts of the monitored pollutants. Power transformations were applied to several of the continuous variables for the regression analysis as their distributions were skewed (Figure 2). To evaluate the combined impact of pollutants each respondent was subsequently ranked based on estimated exposure to NO_2 _and SO_2_, the results of which were in turn summed and ranked to assign an overall exposure rank to individual respondents (1-804) [44]. We split the sample into tertiles of low, medium and high rank in order to compare the determinants of health care access and utilization in spatially representable sub-samples (Figure 1). The number of sub-samples was limited to three because of sample size restrictions and the number of variables included in the analysis. All analyses were carried out with SPSS 17, PASS 11 and ArcGIS 10.0.

## Results

### Sample Characteristics

Table 2 shows the observed frequencies of predictors within the dependant variable classes and the entire sample. Comparing our sample parameters to census data [21] showed that females were slightly over-represented and with respect to age the sample represented an older population than in Sarnia at large. The sample characteristics suggest that the sub-population of Sarnia without a family physician or a regular care provider is disproportionately composed of younger males. This is consistent with reports based on the 2005 Canadian Communities Health Survey data, which found that older people and females used health care services more often than their young and male counter parts [41]. Perceived health status as measured in Statistics Canada's community health profiles [45] was comparable with 57.2 of our sample reporting very good or excellent health versus 59.2% for Sarnia.

**Table 2:** Sample and Statistics Canada Characteristics

	GP Use		GP Access		Sample	Sarnia*
	**Yes**	**No**	**Yes**	**No**		
			
***n***	**187**	**617**	**746**	**58**	**804**	**71 419**

***Percentage***						

**Overall**	23.2	76.8	92.8	7.2		

**Female**	63.1	52.2	55.9	39.7	54.7	52.3

**Low Income^+^**	10.9	8.7	7.9	27.7	9.2	9.0

**No H.S. Diploma^+^**	10.9	12.9	12.1	17.0	12.4	14.8

**Perceived Health (very good/excellent)**	44.4	61.1	57.6	51.7	57.2	59.2

**Housing Tenure (rented)**	44.9	35.3	35.4	65.5	37.6	29.7

**Care Availability**	40.6	44.2	42.0	62.1	43.4	n/a

**GHQ 4+**	30.5	24.8	25.9	29.3	26.1	n/a

**Community Satisfaction**	64.7	60.5	63.7	32.8	61.4	n/a

***Median***						

**Age**	51.3	52.4	53.0	37.5	52.1	48.5

***Mean ± SD***						

**Stress Symptoms (0-7)**	2.7 ± 2.0	1.9 ± 1.7	2.1 ± 1.8	2.1 ± 1.9	2.1 ± 1.8	n/a

**Odour Annoyance (0-10)**	3.8 ± 3.6	2.9 ± 3.2	3.1 ± 3.3	3.3 ± 3.6	3.1 ± 3.3	n/a

*Based on 2006 census [21] and 2010 Health Profile [45]

^+ ^Sample and Census Canada data for ages 25+

The study participants reported having access to a GP at a rate of 92.8%. In Canada the figure changed from 86.4% in 2005 to 84.7% in 2009, but in Ontario the numbers were 91.6% in 2005 and 91.0% in 2009 [46]. A proportion of 23.2% of respondents indicated they had visited or spoken with a GP during the two-week study period. Law et al. found similar rates (19%) of utilization using the same question in Hamilton, Ontario, a city that is also associated with heavy industrialization [16].

Independent samples t-tests showed that respondents who had consulted a GP were on average exposed to higher levels of NO_2 _(*t *= 3.02, *p *< 0.01), and SO_2 _(*t *= 2.79, *p *< 0.01). Also, the results show that respondents without regular care were exposed to higher residential concentrations of NO_2 _(*t *= 2.19, *p *< 0.05) and BTEX (*t *= 2.731, *p *< 0.01). Figure 2 provides box-plot comparisons between pollutant levels within each outcome category. This shows that the median concentrations for all pollutants were higher at the residence of respondents who had consulted a GP or did not have access to regular care.

### Health Care Utilization

Table 3 shows the stepwise logistic regression models for GP use. Mental health measured by the GHQ was the only *a priori *variable that did not contribute to the initial model, while sex (females), age, chronic disease, and GP access significantly increased the odds of GP consultations. These variables remained significant predictors of GP use throughout the analysis, except that gender was insignificant when odour annoyance and community satisfaction were introduced into the model. This could be due to females reporting significantly higher levels of odour annoyance (Anova *F *= 25.05, *df *= 1, *p *< 0.001). The effect of odour annoyance disappeared when pollution related stress symptoms were entered into the model and females also reported significantly higher levels of stress.

**Table 3:** Stepwise Logistic Regression Model for GP Use

	*A priori*	SES	Health & Behaviour	Community Context	Pollution Health Effects (CI)	
	
Sex (Female)	1.514*	1.532*	1.530*	1.426	1.295	(0.893-1.878)
**Age (18-24)**						

**25-44**	2.953*	3.790*	3.560*	3.313*	3.690*	(1.201-11.342)

**45-64**	2.398	3.246*	2.932	2.675	3.162*	(1.204-9.763)

**65+**	2.339	3.100*	3.147*	3.047	4.306*	(1.330-13.938)

**Chronic Conditions**	1.193***	1.194***	1.210***	1.207***	1.131*	(1.020-1.256)

**GHQ 4+**	1.225	1.194	1.199	1.177	1.046	(0.696-1.574)

**GP Access**	2.705*	3.235*	3.388*	2.978*	3.091*	(1.141-8.374)

**Housing Tenure**		1.857***	1.819**	1.844**	1.866**	(1.279-2.723)

**Smoking**			1.598*	1.608*	1.581*	(1.051-2.378)

**Medical Check-up**			1.611*	1.546*	1.622*	(1.060-2.484)

**Community Satisfaction**				1.514*	1.625*	(1.088-2.428)

**Odour Annoyance**				1.068*	1.052	(0.995-1.113)

**Stress Symptoms**					6.236**	(2.128-18.273)

						

**Diagnostics**						

**Model Likelihood Ratio**	χ^2 ^= 37.48, df = 7, p < 0.001	χ^2 ^= 48.67, df = 8, p < 0.001	χ^2 ^= 58.61, df = 10, p < 0.001	χ^2 ^= 66.69, df = 12 p < 0.001	χ^2 ^= 78.13, df = 13, p < 0.001	

**McFadden R^2^**	0.05	0.06	0.07	0.11	0.11	

**Hosmer-Lemeshow Test**	χ^2^= 12.05, df = 8, p = 0.15	χ^2^= 4.35, df = 8, p = 0.82	χ^2^= 6.80, df = 8, p = 0.56	χ^2^= 16.00, df = 8, p = 0.04	χ^2^= 12.55, df = 8, p = 0.128	

Significance level: * < 0.05, ** < 0.01 *** < 0.001.

The final models show that pollutants have different impacts on GP use. Table 4 shows the parsimonious final model for GP use compared to 4 other models that only differ by having SO_2_, NO_2_, BTEX or odour annoyance individually entered. Higher levels of exposure to NO_2 _and SO_2 _significantly increased the likelihood of seeing a doctor by 16-60% during the 2 weeks that monitoring took place. The results produced from this analysis are not very strong potentially due to the relatively low power of the models that included the different pollutants. Only the model that included odour annoyance had higher power than the final stepwise model. The influence of GP access was similar in the control and NO_2 _models, but diminished with SO_2 _and BTEX. Comparing the odds of other variables between the control and pollutant models revealed that the contribution of age and stress also changed markedly. Specifically, there was an increased influence of stress with SO_2 _that attenuated the impact of age. The Wald statistics confirmed that stress proxy made a higher contribution to the model controlling for SO_2 _than NO_2_. Interestingly, we also observed that the impact of smoking on GP use decreased with NO_2 _and BTEX but became insignificant when controlling for SO_2_.

**Table 4:** Logistic Regression Models of GP Use with NO_2_, SO_2_, BTEX and Odour Annoyance

	*No Pollution*	NO_2_	SO_2_	BTEX	Odour Annoyance
		1.155*	1.611*	1.407	1.060*

**Age (18-24)**					

**25-44**	3.765*	3.625*	2.821*	2.969*	3.707*

**45-64**	3.234*	3.195*	2.459*	2.622	3.115*

**65+**	4.242*	4.206*	3.218*	3.485*	4.390*

**Chronic Conditions**	1.139*	1.128*	1.134*	1.134*	1.133*

**GP access**	4.318**	4.516**	3.371*	3.432*	3.150*

**Housing Tenure**	1.932*	1.703**	1.778**	1.858***	1.879***

**Smoking**	1.613*	1.564*	1.471	1.529*	1.581*

**Medical Check-up**	1.679*	1.661*	1.603*	1.640*	1.633*

**Community Satisfaction**	1.536*	1.535*	1.604*	1.599*	1.639*

**Stress Symptoms**	8.523***	8.826***	9.126***	8.946***	7.402***

					

**Diagnostics**					

**Model Likelihood Ratio**	χ^2 ^= 74.81, df = 10, p < 0.001	χ^2 ^= 80.42, df = 11, p < 0.001	χ^2 ^= 76.11, df = 11, p < 0.001	χ^2 ^= 73.63, df = 11 p < 0.001	χ^2 ^= 78.21, df = 11 p < 0.001

**McFadden R^2^**	0.09	0.10	0.10	0.09	0.10

**Hosmer-Lemeshow Test**	χ^2^= 11.79, df = 8, p = 0.16	χ^2^= 12.58, df = 8, p = 0.12	χ^2^= 9.76, df = 8, p = 0.28	χ^2^= 9.88, df = 8, p = 0.27	χ^2^= 14.85, df = 8, p = 0.06

**Correctly Classified (%)**	76.7	77.5	77.5	77.0	76.8

Significance level: * < 0.05, ** < 0.01 *** < 0.001.

Respondents who reported higher satisfaction with their community were approximately 50% more likely to have seen or spoken with their family doctor in the control and all pollution models. We looked to the models measuring access for a possible chain relationship, but entry of the community satisfaction variable into the access model resulted in over-fitting. However, bivariate analysis revealed that there was a significant and directional level of association between access and the ordinal measure of community satisfaction as the dependant variable (Somers' D = 0.31, *p *≤ 0.001). This suggests that community satisfaction is influenced by access to regular care that subsequently affects utilization.

### Access to Primary Health Care

All *a priori *(sex, age, income, unmet health care needs and self-rated health) variables were significant and together provided a model of similar strength to the final GP use models (Table 5). When SES and health related measures were entered into the stepwise procedure the influence of sex and health care needs changed significantly. Males were almost twice as likely as females to have no family physician after SES variables were included, but this effect disappeared with the entry of health and behavioural measures. This was likely due to higher rates of back-pain, joint-pain and easy bruising among women in our sample. Older groups of the sample were more likely to have GP access and this effect remained significant throughout the analysis. Removal of the variable measuring medical check-ups rendered age an insignificant predictor within the entire sample, but in the models that compared high, medium and low exposure sub-samples, only respondents aged 45-64 in the high rank sample had significantly better primary care access.

**Table 5:** Stepwise Logistic Regression Model for GP Access (no regular care)

	*A priori*	SES	Health & Behaviour (CI)	
	
Sex (female)	1.993*	1.865*	1.596	(0.869-2.932)
**Age (18-24)**	**	*	*	

**25-44**	.904	1.199	1.388	(0.532-3.681)

**45-64**	.387*	.540	.648	(0.233-1.802)

**65+**	.280*	.313*	.367	(0.113-1.194)

**Low Income**	4.043***	2.878**	3.001**	(1.411-6.384)

**Unmet Needs**	3.580***	3.904***	4.313***	(2.116-8.790)

**Self-Rated Health**	2.722*	3.426*	3.308*	(1.270-8.616)

**Education**		2.619*	2.575*	(1.097-6.048)

**Housing Tenure**		2.563**	2.458**	(1.275-4.739)

**Control Symptoms**			1.891*	(1.004-3.561)

**Medical Check-ups**			1.719	(0.929-3.182)

**Care Availability**			2.268**	(1.240-4.149)

			

**Diagnostics**				

**Model Likelihood Ratio**	χ^2 ^= 46.38, df = 7, p < 0.001	χ^2 ^= 61.19, df = 9, p < 0.001	χ^2 ^= 75.09, df = 12, p < 0.001	

**McFadden R^2^**	0.12	0.16	0.19	

**Hosmer-Lemeshow Test**	χ^2^= 6.25, df = 7, p = 0.51	χ^2^= 9.07, df = 8, p = 0.34	χ^2^= 6.62, df = 8, p = 0.58	

Significance level: * < 0.05, ** < 0.01 *** < 0.001.

Analysing the exposure ranked sub-samples identified notable relationships between air pollution and heath care. The low exposure sub-sample had the same power as the full sample followed by the high and medium rank samples. High exposure respondents faced stronger barriers to regular primary care when compared to those in lower pollution areas based on several of our independent variables. For instance, we observed that the LICO only predicted access significantly within the medium and high exposure zones and not the low exposure area (Figure 1). Also, respondents living in rental housing within the high exposure area were more likely to lack GP access (OR: 6.02) than low exposure respondents (OR: 4.29) and medium exposure respondents (OR: 2.24). Further analysis of these findings by age distribution showed that there was a significant interaction between housing tenure and exposure rank groups (*F *= 6.96, *df *= 2, *p *< 0.01), possibly owed to the clustering of retirement homes and post-secondary schools. Significant influences of location and wait-times for health care services were restricted to the medium and high exposure sub-samples. Although respondents who spent more than 20 minutes travelling and/or waiting for care were twice as likely to lack regular care overall, this OR was 4.5 in the medium rank, 3.2 in the high rank and non-significant in the low exposure area. The control symptoms did not significantly contribute to GP access in any of the exposure zone sub-samples.

Residual analysis identified a small number of outlying cases with high leverage or influence (DfBeta) values, but the variable coefficients or their significance did not notably differ when these outliers were removed from the analysis. We present McFadden R^2^, or rho-square values, which between 0.2-0.4 represent a very good fit of the model. The pseudo R^2 ^values and model scores indicated that the GP access models were in general stronger than for GP use. This is because it is much more difficult to capture the innumerable reasons for visiting a GP [47] versus the population characteristics associated with health care access. The Hosmer-Lemeshow chi-square statistics indicated that the goodness of fit in the final models were satisfactory.

## Discussion

The study found that multiple dimensions of health along with environmental, socioeconomic, demographic, and health service spatiality predicted GP access and utilization in Sarnia. The importance of differentiating access and utilization, and furthermore taking a closer look at how they interact is exemplified in a high exposure environment. Within the context of compromised environmental quality, our findings demonstrate that neighbourhoods, individual and environmental characteristics can interact to predict access and utilization of health care. Besides the apparent impact of access to regular care on community satisfaction, our analysis revealed that the *a priori*, SES, health care needs and other health related variables were significantly associated with GP access. These results confirm findings from previous studies in Canada and abroad [48]. For instance, the findings here support the OMA's report [4] on the increased cost of health care due to air pollution in the Sarnia region. The results demonstrate that there are significant and specific impacts of different pollutants and their spatial distributions on primary care access and utilization.

A previous study in Sarnia found that high annoyance scores were significantly related to both NO_2 _and SO_2 _levels [35]. Other studies have found associations between NO_2_, cardiac autonomic dysfunction and increased blood pressure, which are also symptoms of stress and can lead to cardiovascular disease [49,50]. NO_2 _can also increase the risk of respiratory tract infections through interaction with the immune system and SO_2 _contributes to respiratory problems for both healthy subjects and those with pulmonary disease [51]. Increased likelihood of GP use from smoking cigarettes was attenuated by NO_2 _and BTEX, and the effect became insignificant with SO_2 _in the model. Therefore, we suggest that NO_2_, BTEX and SO_2 _in Chemical valley increase health care utilization because of their effects on respiratory and cardiovascular diseases prominently featured in the area [20]. Thompson et al. [52] reported that daily fluctuations in benzene concentrations predicted acute asthma emergency admissions of children in Belfast, Northern Ireland, but our analysis showed no direct significant relationship between BTEX and our outcomes. This may be because the predicted distribution of these compounds when taken together does not reveal their individual impacts since their modes of dispersion and the way people respond to them differ. Most health effects associated with VOCs are observed over longer periods of time.

Our results suggest that low SES and stress enhanced by odour annoyance may be compounding health care demands, possibly due to increased susceptibility to adverse pollution impacts (e.g., compromised immune system) [53]. This 'double burden of deprivation' has been identified in studies conducted in Worcester, U.S.A., and Montreal, Canada [15,54]. We observed that the low income only predicted access significantly within the medium and high exposure zones and not the low exposure area. This finding signifies a worrisome interaction between burdens of social and environmental stress, and access to primary health care services. We also found that high exposure ranked respondents were more likely than low ranked respondents to lack GP access if renting, which provides further support for low SES being a barrier to health care.

The role of odour annoyance as a stressor in Sarnia is consistent with work by Shusterman et al. [55] who found that odour mediated mechanisms and annoyance contribute to how people judge and cope with air quality, and furthermore provide important diagnostic information in appraising the potential threats to health and well-being. Research on beliefs regarding toxicity of environmental pollution suggests that "if environments smell bad, they're probably damaging to health" [56] or at the very least, they may reinforce annoyance. Consistent with earlier studies [57], we found females were more likely to report high odour annoyance than their male counter parts. This could be because of gender differences in cognitive and affective processes [57].

Negative perceptions about the environmental health of one's neighbourhood can influence health outcomes along with predisposing determinants of health [58,59]. Consequently, perceived personal susceptibility and severity of health threats are modified by psychosocial factors, which thereafter can influence compliance with medical recommendations and perceived benefits of preventive action [60]. Benefits of access to regular care are not only preventive, but also associated with the responsibility placed on health care providers to inform and educate their patients about hazards of air pollution [61]. It is therefore particularly concerning to find that residents in Sarnia who live in high exposure areas spend more time travelling and waiting for GP consultations. This may be due to primary care providers in Sarnia locating their practices in less polluted areas, thus leaving those in highly polluted areas to travel long distances to seek care. Further research is required to determine the apparent challenges of delivering primary health care services to these areas and their populations.

Although levels of estimated exposure for respondents in the current study were within provincial guidelines, our monitoring methods were not able to capture the impact of individual release events of airborne toxins or "bad air days"; they are not uncommon and are occasionally accompanied by warning sirens and emergency response guides that demand individual coping mechanisms [62]. We can assume that residents at risk from chronic exposure are the same residents at most risk from these events. Residential areas within the high rank zone of Figure 1 provides target areas for improving health care delivery as population characteristics in this zone predicted inequitable access and coincide with high NO_2 _and SO_2 _concentrations that increased the likelihood of utilizing services.

There are a few limitations to this analysis that are worth noting. We note that our conceptualization of access related to a source of regular care at the time of the study and did not account for barriers to acquiring a family doctor or the fact that some people do not attempt to find regular care. Our measure of access did not include alternative sources of care such as walk-in clinics, Telehealth Ontario (free telephone consultation with a registered nurse) and nurse practitioners, and these providers arguably represent important points of access for certain deprived populations identified in this study. A study that looked at access to family physicians in Southwestern Ontario found that of the 9.1% of the population that did not have regular care, 55% used walk-in clinics and 13% used emergency rooms as their source of care [63]. The study was also limited by lacking longitudinal measures of health and SES as the study used a cross-sectional design. O'Neill et al. [64] suggest studies on air pollution that include SES measures consider how they change through the course of life. They also propose that exposure assessment include the effect of daily movement, which our study design did not permit. Furthermore, we found that our model predicting health care utilization was relatively weak, but there were nonetheless significant associations with air pollution that have potential implications for policy.

## Conclusions

The aim of the study was to examine the relationship between spatially sensitive measures of air pollution and general practitioner access and utilization in a high exposure environment. Our results provide further support to the pursuit of better access to regular primary care in communities faced with environmental challenges. The Ontario Medical Association projects that increased numbers of premature deaths, hospital admissions and emergency visits due to air pollution in Lambton County, which includes Sarnia and smaller communities in the surrounding area, will increase to $6 million in lost productivity and $8 million in additional health care costs by 2026. We conclude that some of these costs can be avoided by ensuring equitable access to primary care for residents most severely affected by air pollution.

## Abbreviations

AOC: Area of Concern; BTEX: Benzene, toluene, ethylbenzene, xylene; CI: Confidence Interval; GP: General Practitioner; LUR: Land Use Regression; NO_2_: Nitrogen dioxide; OMA: Ontario Medical Association; OR: Odds Ratio; SES: Socioeconomic Status; SO_2_: Sulphur dioxide; VOC(s): Volatile Organic Compound(s).

## Competing interests

The authors declare that they have no competing interests.

## Authors' contributions

THO and IL conceived the study and were involved in interpretation of the results and preparation of the manuscript. DA and KG were involved in the analysis and all authors have read and approved the final manuscript.

## References

[B1] Glouberman S Millar J Evolution of the determinants of health, health policy, and health information systems in Canada Am J Public Health 2003 93 388 392 10.2105/AJPH.93.3.388 12604478 PMC1447749

[B2] Frohlich KL Ross N Richmond C Health disparities in Canada today: Some evidence and a theoretical framework Health Policy 2006 79 132 143 10.1016/j.healthpol.2005.12.010 16519957

[B3] Jerrett M Eyles J Dufournaud C Birch S Environmental influences on healthcare expenditures: An exploratory analysis from Ontario, Canada Journal of Epidemiology and Community Health 2003 57 334 338 10.1136/jech.57.5.334 12700215 PMC1732448

[B4] Ontario MedicalAssociation Illness Costs of Air Pollution (ICAP) - Regional Data for 2005 (with projections to 2026) 2005 http://www.oma.org/phealth/smogmain.htm

[B5] Yip AM Kephart G Veugelers PJ Individual and neighbourhood determinants of health care utilization: Implications for health policy and resource allocation Canadian Journal of Public Health 2002 93 303 307 10.1007/BF03405022 PMC6980116 12154535

[B6] Birch S Eyles J Newbold KB Equitable access to health care: methodological extensions to the analysis of physician utilization in Canada Health economics 1993 2 87 101 10.1002/hec.4730020203 8261040

[B7] Dunlop S Coyte PC McIsaac W Socio-economic status and the utilisation of physicians' services: Results from the Canadian National Population Health Survey Social Science and Medicine 2000 51 123 133 10.1016/S0277-9536(99)00424-4 10817475

[B8] Finkelstein MM Do factors other than need determine utilization of physicians' services in Ontario? Canadian Medical Association Journal 2001 165 565 570 11563208 PMC81414

[B9] Curtis LJ MacMinn WJ Health care utilization in Canada: Twenty-five years of evidence Canadian Public Policy 2008 34 65 87 10.3138/cpp.34.1.065

[B10] Rosenberg MW Hanlon NT Access and utilization: A continuum of health service environments Social Science and Medicine 1996 43 975 983 10.1016/0277-9536(96)00007-X 8888467

[B11] Wellstood K Wilson K Eyles J 'Reasonable access' to primary care: Assessing the role of individual and system characteristics Health and Place 2006 12 121 130 10.1016/j.healthplace.2004.10.010 16338628

[B12] Birch S Eyles J Hurley J Hutchison B Chambers S A Needs-Based Approach to Resource Allocation in Health Care Canadian Public Policy/Analyse de Politiques 1993 19 68 85 10.2307/3551791

[B13] McLafferty SL GIS and health care Annu Rev Public Health 2003 24 25 42 10.1146/annurev.publhealth.24.012902.141012 12668754

[B14] Andersen RM Revisiting the behavioral model and access to medical care: does it matter? Journal of Health and Social Behavior 1995 36 1 10 10.2307/2137284 7738325

[B15] Crouse DL Ross NA Goldberg MS Double burden of deprivation and high concentrations of ambient air pollution at the neighbourhood scale in Montreal, Canada Social Science and Medicine 2009 69 971 981 10.1016/j.socscimed.2009.07.010 19656603

[B16] Law M Wilson K Eyles J Elliott S Jerrett M Moffat T Luginaah I Meeting health need, accessing health care: the role of neighbourhood Health Place 2005 11 367 377 10.1016/j.healthplace.2004.05.004 15886144

[B17] Fuchs VR Frank SR Air pollution and medical care use by older Americans: a cross-area analysis Health Aff (Millwood) 2002 21 207 214 10.1377/hlthaff.21.6.207 12442858

[B18] Starfield B Shi L Macinko J Contribution of Primary Care to Health Systems and Health Milbank Q 2005 83 457 502 10.1111/j.1468-0009.2005.00409.x 16202000 PMC2690145

[B19] Gwynn RC Thurston GD The burden of air pollution: impacts among racial minorities Environ Health Perspect 2001 109 Suppl 4 501 506 11544154 10.1289/ehp.01109s4501 PMC1240572

[B20] Fung KY Luginaah IN Gorey KM Impact of air pollution on hospital admissions in Southwestern Ontario, Canada: Generating hypotheses in sentinel high-exposure places Environ Health 2007 6 18 10.1186/1476-069X-6-18 17612400 PMC1929065

[B21] Statistics Canada Sarnia, Ontario, 2006 Community Profiles 2007 2006 Census. http://www12.statcan.ca/census-recensement/2006/dp-pd/prof/92-591/index.cfm?Lang=E

[B22] Health Canada Health Data and Statistics Compilation for Great Lakes Areas of Concern: Health Canada 2000

[B23] Van Larebeke NA Sasco AJ Brophy JT Keith MM Gilbertson M Watterson A Sex ratio changes as sentinel health events of endocrine disruption International Journal of Occupational and Environmental Health 2008 14 138 143 18507291 10.1179/oeh.2008.14.2.138

[B24] Mackenzie CA Lockridge A Keith M Declining sex ratio in a First Nation community Environmental Health Perspectives 2005 113 1295 1298 10.1289/ehp.8479 16203237 PMC1281269

[B25] Peduzzi P Concato J Kemper E Holford TR Feinstein AR A simulation study of the number of events per variable in logistic regression analysis J Clin Epidemiol 1996 49 1373 1379 10.1016/S0895-4356(96)00236-3 8970487

[B26] Adler R Vasiliadis A Bickell N The relationship between continuity and patient satisfaction: A systematic review Fam Pract 2010 27 171 178 10.1093/fampra/cmp099 20053674

[B27] Guagliardo MF Spatial accessibility of primary care: concepts, methods and challenges Int J Health Geogr 2004 3 3 10.1186/1476-072X-3-3 14987337 PMC394340

[B28] Roberts RO Bergstralh EJ Schmidt L Jacobsen SJ Comparison of self-reported and medical record health care utilization measures J Clin Epidemiol 1996 49 989 995 10.1016/0895-4356(96)00143-6 8780606

[B29] Atari DO Luginaah IN Assessing the distribution of volatile organic compounds using land use regression in Sarnia, "Chemical Valley", Ontario, Canada Environ Health 2009 8 16 10.1186/1476-069X-8-16 19371421 PMC2679013

[B30] Atari DO Luginaah I Xu X Fung K Spatial variability of ambient nitrogen dioxide and sulfur dioxide in Sarnia, "Chemical Valley," Ontario, Canada Journal of Toxicology and Environmental Health - Part A: Current Issues 2008 71 1572 1581 10.1080/15287390802414158 18850457

[B31] Brauer M Hoek G van Vliet P Meliefste K Fischer P Gehring U Heinrich J Cyrys J Bellander T Lewne M Brunekreef B Estimating long-term average particulate air pollution concentrations: Application of traffic indicators and geographic information systems Epidemiology 2003 14 228 239 12606891 10.1097/01.EDE.0000041910.49046.9B

[B32] Levy JI Houseman EA Ryan L Richardson D Spengler JD Particle concentrations in urban microenvironments Environ Health Perspect 2000 108 1051 1057 10.2307/3434958 11102296 PMC1240162

[B33] Jerrett M Arain A Kanaroglou P Beckerman B Potoglou D Sahsuvaroglu T Morrison J Giovis C A review and evaluation of intraurban air pollution exposure models J Expo Anal Environ Epidemiol 2005 15 185 204 10.1038/sj.jea.7500388 15292906

[B34] Hoek G Beelen R de Hoogh K Vienneau D Gulliver J Fischer P Briggs D A review of land-use regression models to assess spatial variation of outdoor air pollution Atmos Environ 2008 42 7561 7578 10.1016/j.atmosenv.2008.05.057

[B35] Atari DO Luginaah IN Fung K The Relationship between Odour Annoyance Scores and Modelled Ambient Air Pollution in Sarnia, "Chemical Valley", Ontario International Journal of Environmental Research and Public Health 2009 6 2655 2675 10.3390/ijerph6102655 20054461 PMC2790099

[B36] Luginaah IN Martin Taylor S Elliott SJ Eyles JD Community reappraisal of the perceived health effects of a petroleum refinery Soc Sci Med 2002 55 47 61 10.1016/S0277-9536(01)00206-4 12137188

[B37] Goldberg DP The detection of psychiatric illness by questionnaire 1972 London

[B38] Willmott SA Boardman JAP Henshaw CA Jones PW Understanding General Health Questionnaire (GHQ-28) score and its threshold Soc Psychiatry Psychiatr Epidemiol 2004 39 613 617 15300371 10.1007/s00127-004-0801-1

[B39] Statistics Canada Low Income Cut Offs for 2005 and Low Income Measures for 2006 2005 http://www.statcan.gc.ca/pub/75f0002m/75f0002m2007004-eng.pdf

[B40] Dunn JR Housing and inequalities in health: a study of socioeconomic dimensions of housing and self reported health from a survey of Vancouver residents Journal of Epidemiology and Community Health 2002 56 671 681 10.1136/jech.56.9.671 12177083 PMC1732232

[B41] Nabalamba A Millar WJ Going to the doctor Health reports/Statistics Canada, Canadian Centre for Health Information = Rapports sur la santé/Statistique Canada, Centre canadien d'information sur la santé 2007 18 23 35 17441441

[B42] Sanmartin C Ross N Experiencing Difficulties Accessing First-Contact Health Services in Canada: Canadians without regular doctors and recent immigrants have difficulties accessing first-contact healthcare services. Reports of difficulties in accessing care vary by age, sex and region Healthc Policy 2006 1 103 119 19305660 PMC2585333

[B43] Hosmer DW Lemeshow S Applied logistic regression 2000 2 New York: Wiley

[B44] Miller L Xu XH Luginaah I Spatial Variability of Volatile Organic Compound Concentrations in Sarnia, Ontario, Canada Journal of Toxicology and Environmental Health-Part A-Current Issues 2009 72 610 624 10.1080/15287390802706413 19296410

[B45] Statistics Canada Health Profile. Statistics Canada Catalogue No. 82-228-XWE 2010 http://www12.statcan.gc.ca/health-sante/82-228/index.cfm?Lang=E

[B46] Statistics Canada Table 105-3024 - Population reporting a regular family physician, household population aged 15 and over, Canada, provinces and territories, occasional, CANSIM (database) http://cansim2.statcan.gc.ca/cgi-win/cnsmcgi.exe?Lang=E&CNSM-Fi=CII/CII_1-eng.htm

[B47] Ogden J Andrade J Eisner M Ironmonger M Maxwell J Muir E Siriwardena R Thwaites S To treat? To befriend? To prevent? Patients' and GPs' views of the doctor's role Scand J Prim Health Care 1997 15 114 117 10.3109/02813439709018499 9323776

[B48] Lasser KE Himmelstein DU Woolhandler S Access to care, health status, and health disparities in the United States and Canada: Results of a cross-national population-based survey Am J Public Health 2006 96 1300 1307 10.2105/AJPH.2004.059402 16735628 PMC1483879

[B49] Dietrich DF Gemperli A Gaspoz JM Schindler C Liu LJS Gold DR Schwartz J Rochat T Barthélémy JC Pons M Roche F Probst Hensch NM Bridevaux PO Gerbase MW Neu U Ackermann-Liebrich U Differences in Heart Rate Variability Associated with Long-Term Exposure to NO2 Environ Health Perspect 2008 116 1357 1361 10.1289/ehp.11377 18941578 PMC2569095

[B50] Lucini D Di Fede G Parati G Pagani M Impact of chronic psychosocial stress on autonomic cardiovascular regulation in otherwise healthy subjects Hypertension 2005 46 1201 1206 10.1161/01.HYP.0000185147.32385.4b 16203875

[B51] Chen T-Gokhale J Shofer S Kuschner WG Outdoor air pollution: Nitrogen dioxide, sulfur dioxide, and carbon monoxide health effects American Journal of the Medical Sciences 2007 333 249 256 10.1097/MAJ.0b013e31803b900f 17435420

[B52] Thompson AJ Shields MD Patterson CC Acute asthma exacerbations and air pollutants in children living in Belfast, Northern Ireland Arch Environ Health 2001 56 234 241 10.1080/00039890109604447 11480499

[B53] Clougherty JE Kubzansky LD A framework for examining social stress and susceptibility to air pollution in respiratory health Environ Health Perspect 2009 117 1351 1358 19750097 10.1289/ehp.0900612 PMC2737009

[B54] Yanosky JD Schwartz J Suh HH Associations Between Measures of Socioeconomic Position and Chronic Nitrogen Dioxide Exposure in Worcester, Massachusetts Journal of Toxicology and Environmental Health-Part A-Current Issues 2008 71 1593 1602 10.1080/15287390802414307 18850459

[B55] Shusterman D Lipscomb J Neutra R Satin K Symptom prevalence and odor-worry interaction near hazardous waste sites Environ Health Perspect 1991 94 25 30 1954935 10.1289/ehp.94-1567940 PMC1567940

[B56] Williams CW Lees-Haley PR Effect of information about odor on causal ascriptions for illness Percept Mot Skills 1997 85 411 418 10.2466/PMS.85.6.411-418 9347522

[B57] Krewski D Lemyre L Turner MC Lee JEC Dallaire C Bouchard L Brand K Mercier P Public perception of population health risks in Canada: Health hazards and sources of information Hum Ecol Risk Assess 2006 12 626 644 10.1080/10807030600561832

[B58] Wilson K Elliott S Law M Eyles J Jerrett M Keller-Olaman S Linking perceptions of neighbourhood to health in Hamilton, Canada J Epidemiol Community Health 2004 58 192 198 10.1136/jech.2003.014308 14966230 PMC1732692

[B59] Stenlund T Liden E Andersson K Garvill J Nordin S Annoyance and health symptoms and their influencing factors: A population-based air pollution intervention study Public Health 2009 123 339 345 10.1016/j.puhe.2008.12.021 19344922

[B60] Green LW Modifying and Developing Health Behavior Annu Rev Public Health 1984 5 215 236 10.1146/annurev.pu.05.050184.001243 6372810

[B61] Brook RD Franklin B Cascio W Hong Y Howard G Lipsett M Luepker R Mittleman M Samet J Smith SC Tager I Expert Panel on Population and Prevention Science of the American Heart Association Air pollution and cardiovascular disease: a statement for healthcare professionals from the Expert Panel on Population and Prevention Science of the American Heart Association Circulation 2004 109 2655 2671 10.1161/01.CIR.0000128587.30041.C8 15173049

[B62] Atari DO Luginaah I Baxter J "This is the mess that we are living in": residents everyday life experiences of living in a stigmatized community GeoJournal 2010 75 1 18 10.1007/s10708-010-9341-2

[B63] Reid GJ Freeman TR Thind A Stewart M Brown JB Vingilis ER Access to family physicians in southwestern ontario Healthc Policy 2009 5 e187 206 21037821 PMC2805148

[B64] O'Neill MS Jerrett M Kawachi L Levy JL Cohen AJ Gouveia N Wilkinson P Fletcher T Cifuentes L Schwartz J Health, wealth, and air pollution: Advancing theory and methods Environ Health Perspect 2003 111 1861 1870 10.1289/ehp.6334 14644658 PMC1241758

